# Short term resistance training enhanced plasma apoA-I and FABP4 levels in Streptozotocin-induced diabetic rats

**DOI:** 10.1186/2251-6581-13-41

**Published:** 2014-03-04

**Authors:** Alireza Safarzade, Elahe Talebi-Garakani

**Affiliations:** 1 Department of Exercise Physiology, Faculty of Physical Education & Sport Science, University of Mazandaran, Babolsar, Iran

**Keywords:** Resistance training, aP2, A-FABP, apoA-I, Lipid profile, Diabetic rats

## Abstract

**Background:**

Type 1 diabetes mellitus is associated with a high risk for early atherosclerotic complications. Altered lipids and lipoprotein metabolism in chronic diabetes mellitus is associated with pathogenesis of atherosclerosis and other cardiovascular diseases. The aim of this study was to investigate the effects of 4 weeks resistance training on plasma lipid profile, fatty acid binding protein (FABP) 4 and apolipoprotein (apo) A-I levels in type 1 diabetic rats.

**Methods:**

Thirty two male Wister rats (12–14 weeks old) were randomly divided into four groups: non-diabetic control; non-diabetic trained; diabetic control; diabetic trained. The rats in training groups were subjected to a resistance training program (3 days/wk, for 4 wk) consisted of climbing a ladder carrying a load suspended from the tail.

**Results:**

Diabetic inducing increased plasma apoA-I and decreased FABP4 levels compared with non-diabetic control group (respectively, P = 0.001 & P = 0.041). After 4 weeks’ resistance training, plasma levels of apoA-I and FABP4 in the diabetic trained rats were significantly higher compared with the diabetic control group (respectively, P = 0.003 & P = 0.017). Plasma HDL-C level in diabetic trained group was higher than diabetic control group (P = 0.048). Liver triglycerides concentrations were significantly lower in both trained (non-diabetic and diabetic) groups compared with their control groups (respectively, P = 0.041 and P = 0.002).

**Conclusion:**

These data indicated that resistance training may be an efficient intervention strategy to increase plasma apoA-I, HDL-C and FABP4 concentrations, along with decreases liver triglycerides in streptozotocin induced diabetic rats. Further research is needed to elucidate physiological significance of circulating FABP4 levels.

## Introduction

The incidence of diabetes mellitus is increasing worldwide, and data from recent studies suggest that the increase is exponential rather than linear [1]. This metabolic disease is associated with hyperglycemia, which leads to many pathological changes, including peripheral neuropathy, nephropathy, retinopathy, microvascular lesions, liver disease and a variety of diseases that diminish the quality of life and life expectancy of the patients [2,3]. Type 1 diabetes mellitus is associated with a high risk for early atherosclerotic complications. In this patients risk of coronary heart disease is 4 to 8 more excessive than general population [4]. Lipids play an important role in maintaining the integrity of biomembrane structure and functions. Altered lipids and lipoprotein metabolism in chronic diabetes mellitus is associated with pathogenesis of atherosclerosis and other cardiovascular diseases [5]. Abnormalities in plasma lipids and lipoprotein patterns due to defect in insulin insufficiency has been well documented in both type I and type II diabetes mellitus [6]. In these patients, classic lipid profile may be normal but patient is at increased risk of atherosclerosis. Measurement of apolipoproteins in diabetic patients may be helpful in diabetic patients at risk of cardiovascular diseases [7]. Apolipoprotein A-I (apoA-I) is the major protein of high-density lipoprotein (HDL) particles and thus provides an indication of the number of anti-atherogenic particles, but without a simple one-to-one relationship in this case [8].

Fatty acid binding protein (FABP) 4 (or adipocyte FABP or adipocyte protein 2 [aP2]) serves as a carrier protein for fatty acids and other lipophilic substances between extra- and intracellular membranes, and has been introduced as a novel adipokine that is secreted from mature adipocytes into bloodstream [9]. Although the function of serum FABP4 has not yet been elucidated, results from recent publications suggest a systemic effect of FABP4 on peripheral tissues [10]. Previous studies indicated that circulating FABP4 levels was increased in overweight and obese subjects compared with lean controls and was associated with markers of insulin resistance and obesity [11-13]. Furthermore, it was demonstrated that serum FABP4 levels are increased in patients with gestational diabetes mellitus, preeclampsia, and renal dysfunction [14-16]. Paradoxically, lower circulating levels of FABP4 found in chronic pancreatitis patients [17], and lean compared with obese type 1 diabetic patients [18].

In addition to dietary control, physical exercise has been heavily used as a non-pharmaceutical treatment for the control and reduction in abnormal levels of circulating lipids and glucose in individuals with metabolic dysfunctions such as diabetes [19]. Although the effect of chronic aerobic exercise upon lipid profile has been demonstrated, few studies have investigated this effect under resistance exercise conditions in subjects with type 1 diabetes. Among the exercise interventions, resistance training has been shown to be efficient in decreasing the adipose tissue depots in rats [20-22]. Resistance training also improves body composition, physical fitness, quality of life, and lipid profile [23,24].

Considering the well-established capacity of resistance training for increasing both muscle mass and strength, as well as improving the lipid profile, adiposity and obesity-associated inflammation, we have sought to investigate the effects of a resistance training program, mainly composed of concentric contractile force production on plasma FABP4, apoA-I and lipids profile in plasma and liver tissue in Streptozotocin-induced diabetic rats.

## Materials and methods

### Animals

Thirty two male Wister rats (289 ± 23 g, 12–14 weeks old) were utilized at the beginning of this study. Animals were housed in cages under controlled light/dark (12/12 h) and temperature (22 ± 2°C) conditions, and were provided with standard food and water *ad libitum*. The animals were acclimated to their living conditions for 1 week. Then they were randomly separated into one of four experimental groups (N = 8 in each group): non-diabetic control (non-DC); non-diabetic trained (non-DT); diabetic control (DC); diabetic trained (DT). The exercise groups undertook a 4 weeks resistance training program. All methods used were approved by the Ethics Committee of the School of Medical Sciences, Tarbiat Modares University.

### Induction of diabetes

Diabetes was induced by a single intraperitoneal injection of streptozotocin (STZ) at a dose of 55 mg/kg (Sigma-Aldrich, St. Louis, MO). STZ was dissolved (20 mg/ml) in a cold 0.1 M citrate buffer (pH 4.5). Non-diabetic rats were injected with a similar volume of citrate buffer only. Five days after the STZ injection, blood glucose concentration was measured using tail vein blood samples obtained from rats following overnight fasting. A blood glucose level > 14 mmol/L was considered indicative of diabetes.

### Resistance training protocol

Resistance training was accomplished with the use of a 1-m ladder inclined at 80°. There were 26 rungs evenly spaced on the ladder. Before inducing diabetes, rats were familiarized with the ladder by practicing climbing the ladder from the bottom to the top of ladder without any additional weight. Rats were positioned at the bottom of climbing apparatus and motivated to climb the ladder by touching and grooming of the tail. When the rats reached the top of the ladder, they were allowed to rest in a simulated home. Resistance training was initiated 7 days after injection of STZ using weights that were attached to the base of tail with an adhesive tape and a clip. All animals were weighed every 4 days to monitor weight gains and, for the resistance trained animals, to determine the amount of weight to append to their tails for the remainder of the week. The training program was divided into two parts: the preliminary phase of 2 weeks duration followed by a sustained non-incremental resistance training intervention phase of 2 weeks duration with the loading equivalent to 100% body mass (BM). In the preliminary phase, the rats were adapted to climbing the ladder with progressive loading on each successive training day. Both training groups of rats performed 6 repetitions ascending the ladder interspersed with 1:00 minute rest intervals. A second set was performed after a 3:00 minutes rest. On the first day, rats trained with the equivalent of 30% BM as load appended to their tail (6 repetitions/2 sets). On the second day the training load was increased to 50% BM (6 reps/2 sets), and on the third day an additional set of repetitions was undertaken with 50% BM (6 reps/3 sets). Thereafter the training load was gradually increased until the seventh day when the training load reached 100% BM. In the final 2 weeks of the resistance training phase, the rats continued to train with 100% BM, 6 repetitions per set, 3 sets per day, and 3 days per week until the end of week 4. Each repetition lasted ~ 4–6 s. The resistance training protocol was adapted from Lee and Farrar [25] to meet the needs and focus of the current research. Warming-up and cooling down consisted of 2 repetitions climbing the ladder without weights appended to the tail, immediately pre and post each training session. Non-trained (controls) rats were handled on the same days and times as the trained groups in order to minimize any stress attributable to handling.

### Euthanasia and sampling

To minimize any residual effect of the last training bout, 48 h after the last training session, rats were anesthetized intraperitoneally with a mixture of Ketamine (50 mg/kg) and Xylazine (3–5 mg/kg). Rats were sacrificed between 9:00 and 12:00 a.m. after fasting overnight. The abdominal cavity was opened following the median line of the abdomen and approximately 6 ml of blood was collected from the abdominal vena cava and was collected in tubes containing EDTA. Blood was centrifuged (3000 rpm; 4°C; 15 min) and the plasma was kept for further analyses.

Samples of liver was quickly collected from the animals and immediately frozen in liquid nitrogen. The flexor hallucis longus (FHL) muscle (the major muscle recruited in climbing activity), free from surrounding tissues, was rapidly dissected from the right hind-limb, weighed, and immediately frozen in liquid nitrogen. The samples were placed into tubes with buffer containing 0.9% NaCl, 50 mM Tris–HCl, 12 μM leupeptin. They were then homogenized with a homogenizer (Potter-Elvejheim) set at 800 rpm for 20 seconds. After this procedure, the samples were centrifuged at 4.000 rpm for 10 minutes. The supernatant was extracted to determine the triglycerides and cholesterol. All samples were stored at -78°C until analyses were performed.

### Biochemical measurements

Plasma glucose was determined by an enzymatic (GOD-PAP, Glucose Oxidase-Amino Antipyrine) colorimetric method (Pars Azmoun, Tehran, Iran). ELISA kits specific for rat studies were used to determine plasma insulin (Mercodia AB, Uppsala, Sweden), apoA-I (Cusabio Biothech, Wuhan, China), and FABP4 (Cusabio Biothech, Wuhan, China) concentrations following the manufacturer’s instructions. Plasma high-density lipoprotein cholesterol (HDL-C) was determined by direct Colorimetric Method (Randox, Antrim, UK), total Triglycerides (TG) by enzymatic (Glycerol-3-Phosphate Oxidase) colorimetric method (Pars Azmoun, Tehran, Iran), total Cholesterol (TC) by enzymatic (Cholesterol Oxidase-Amino Antipyrine) colorimetric method (Pars Azmoun, Tehran, Iran), and non-esterified fatty acids (NEFA) concentration by a Colorimetric Method (Randox, Antrim, UK) following the manufacturer’s instructions. The procedure of Friedewald et al. was used to estimate low-density lipoprotein cholesterol (LDL-C) according to the following formula:

$$\mathrm{LDL}\;\mathrm{cholesterol}=\mathrm{total}\;\mathrm{cholesterol}-\mathrm{HDL}\;\mathrm{cholesterol}-\left(\mathrm{Triglycerides}/5\right).$$

### Statistical analysis

All statistical analyses were undertaken using the SPSS program, version 16.0, for Windows. All values were checked for normality using Kolmogorov-Smirnov test. Paired sample Student’s t test was used to test the difference between pre and post of some parameters (for continuous variables). Comparisons between groups were made using one-way analysis of variance (ANOVA) and LSD’s test. Correlations analyses were performed using Pearson’s method to examine the simple relationships between plasma apoA-I and FABP4 concentration and selected variables in diabetic and non-diabetic groups. All data were expressed as mean ± SD and *P* value ≤0.05 was considered statistically significant.

## Results

**Figure 1:**
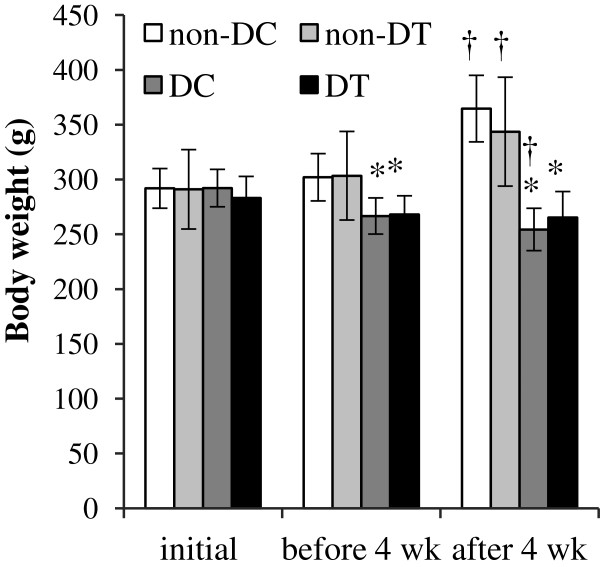
**Body weights (g) of rats in non-diabetic control (non-DC), non-diabetic trained (non-DT), diabetic control (DC), and diabetic trained (DT) rats.** * *P < 0.05*; significantly different from Non-diabetic control. † *P < 0.05*; significantly different from before 4 weeks.

All of the trained animals successfully completed the 4 weeks of resistance training. The results of body weights changes are shown in Figure 1. Initial body weights between the groups were not significantly different in this study. Five days after the STZ injection (before 4 weeks of resistance training), weight loss was shown in diabetic rats (i.e., DC and DT). Final body weights of diabetic control and trained groups were significantly lower than non-diabetic groups (P < 0.001). Body weight of diabetic control group significantly reduced after 4 weeks compared with before the 4 weeks of training program (P = 0.019). But in diabetic trained group we did not find any significant difference between before and after 4 weeks of resistance training program. The wet weight of the FHL muscle in diabetic rats was significantly lower than in non-diabetic groups (P < 0.05) (Table 1). The weight of the FHL muscle was significantly greater in trained diabetic rats compared with sedentary diabetic rats (P = 0.026). Also, the ratio of FHL weight per whole body weight in diabetic trained rats was significantly higher than in sedentary diabetic rats (P = 0.002).

**Table 1:** Plasma Concentrations of glucose, insulin, lipid profile, and liver cholesterol and triglycerides

	**Non-diabetic control**	**Non-diabetic trained**	**Diabetic control**	**Diabetic trained**
Glucose (mg/dl)	126 ± 5.8	130 ± 6.5	338 ± 29.9^*^	346 ± 15.6^*^
Insulin (μg/L)	0.43 ± 0.11	0.45 ± 0.07	0.17 ± 0.07^*^	0.16 ± 0.06^*^
Total cholesterol (mg/dl)	75.4 ± 9.7	79.6 ± 10.9	73.9 ± 11.5	80.7 ± 7.8
Triglycerides (mg/dl)	64.6 ± 10.9	60.4 ± 7.2	69.0 ± 14.6	70.7 ± 7.0
HDL- cholesterol (mg/dl)	27.4 ± 1.5	27.6 ± 3.3	26.3 ± 4.1	30.4 ± 5.5†
LDL- cholesterol (mg/dl)	36.4 ± 7.9	39.9 ± 10.1	33.8 ± 11.1	36.2 ± 6.1
Non-esterified fatty acid (mM)	0.78 ± 0.14	0.83 ± 0.17	0.77 ± 0.13	0.82 ± 0.14
Liver cholesterol (mg/g)	4.1 ± 2.31	2.8 ± 1.07	5.0 ± 1.66	3.8 ± 1.62
Liver triglycerides (mg/g)	34.6 ± 4.5	28.3 ± 5.7^*^	31.4 ± 7.4	21.4 ± 4.6^*†^
FHL (mg)	508.2 ± 35.2	478.5 ± 61.7	286.2 ± 46.1^*^	334.0 ± 37.6^†^
FHL/body weight (mg/g)	1.40 ± 0.09	1.40 ± 0.10	1.13 ± 0.11^*^	1.28 ± 0.10^†^

The values are presented as mean ± standard deviation of eight animals per group. *P < 0.05; significantly different from Non-diabetic control. ^†^*P < 0.05*; significantly different from Diabetic control.

**Figure 2:**
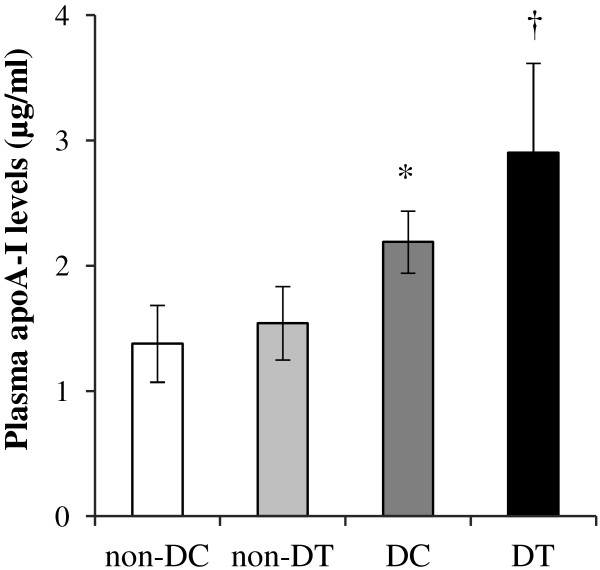
**Plasma concentrations of apoA-I in non-diabetic control (non-DC), non-diabetic trained (non-DT), diabetic control (DC), and diabetic trained (DT) rats.** The values are presented as mean ± standard deviation of 8 animals per group. *P < 0.05; significantly different from Non-diabetic control. † *P < 0.05*; significantly different from Diabetic control.

**Figure 3:**
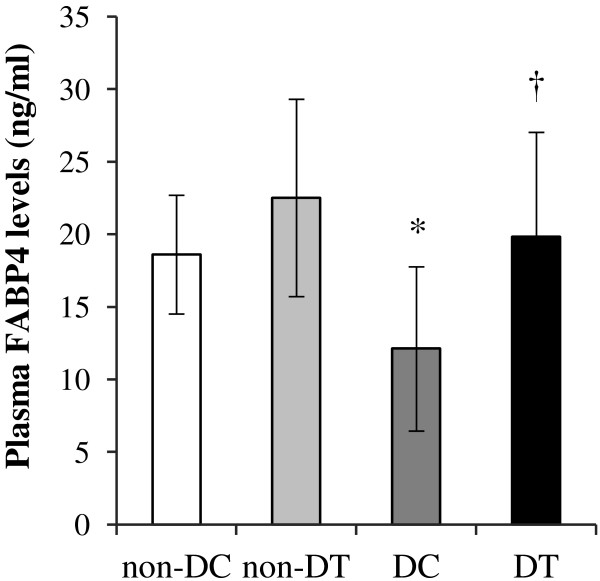
**Plasma concentrations of FABP4 in non-diabetic control (*non-DC*), non-diabetic trained (*non-DT*), diabetic control (*DC*), and diabetic trained (*DT*) rats.** The values are presented as mean ± standard deviation of 8 animals per group. *P < 0.05; significantly different from Non-diabetic control. † *P < 0.05*; significantly different from Diabetic control.

Both diabetic (control and trained) groups had significantly higher plasma glucose and lower insulin concentrations than non-diabetic (control and trained) groups (Table 1). Four weeks’ resistance training did not significantly affect the plasma glucose and insulin concentrations in diabetic and non-diabetic groups. Plasma levels of total cholesterol, triglycerides, NEFA, and LDL-C did not differ between all groups. After 4 weeks resistance training plasma HDL-C level in diabetic trained group was higher compared with diabetic control group (P = 0.048). The statistical analysis did not show a significant difference in liver cholesterol concentrations between all groups. Liver triglycerides concentrations were significantly lower in non-diabetic trained and diabetic trained groups compared with their control groups (respectively, P = 0.041 and P = 0.002).Diabetic inducing increased plasma apoA-I (P = 0.001) levels compared with non-diabetic control rats (Figure 2). After 4 weeks’ resistance training, plasma levels of apoA-I in the diabetic trained rats was significantly higher compared with the diabetic control group (P = 0.003).Plasma level of FABP4 was significantly (P = 0.041) lower in control diabetic rats compared with non-diabetic control group (Figure 3). Four weeks’ resistance training significantly increased plasma FABP4 levels compared with diabetic control group (P = 0.017).

We examined the correlation between plasma apoA-I and FABP4 concentrations and body weights and FHL muscle mass in both diabetic and non-diabetic rats (Table 2). In diabetic rats plasma apoA-I and FABP4 concentrations correlated positively with FHL muscle mass and ratio of FHL per body weight. However in non-diabetic group plasma apoA-I and FABP4 concentrations were not significantly correlated with these parameters. Plasma FABP4 levels in diabetic rats positively correlated with body weight, but statistically was not significant (r = 0.496, *P* = 0.051).

**Table 2:** Pearson correlation between plasma apoA-I and FABP4 levels and muscle and body weights parameters in both diabetic and non-diabetic rats

	**apoA-I**		**FABP4**	
	**Diabetic (n = 16)**	**Non- diabetic (n = 16)**	**Diabetic (n = 16)**	**Non- diabetic (n = 16)**
	***r (P *** **value)**	***r (P *** **value)**	***r (P *** **value)**	***r (P *** **value)**
Final body weight (g)	0.396 (0.129)	- 0.099 (0.717)	0.496 (0.051)	- 0.109 (0.689)
Weight gain (g)	0.455 (0.077)	- 0.485 (0.057)	0.461 (0.072)	- 0.023 (0.933)
FHL (mg)	0.577 (0.019)	0.056 (0.836)	0.683 (0.004)	0.143 (0.597)
FHL/body weight (mg/g)	0.544 (0.029)	0.274 (0.305)	0.647 (0.007)	0.437 (0.090)

Weight gain = Final body weight- Initial body weight.

## Discussion

In this experimental study, we found that hyperglycemia in streptozotocin- induced diabetic rats was associated with higher plasma apoA-I and lower FABP4 levels than non-diabetic control rats. Also, 4 weeks’ resistance training increased plasma FABP4 and apoA-I levels in diabetic trained rats when compared with diabetic control group.

ApoA-I, the major protein of HDL, is produced in both the liver and the intestine and is secreted into the plasma as a lipid-poor apolipoprotein. ApoA-I production would stimulate physiological formation of new HDL particles, which would carry out their biological activity and is supported by observations from animal models that transgenic expression of apoA-I is atheroprotective [26].

In the present study, we found that plasma levels of apoA-I in diabetic control group was higher compared with the non-diabetic control group. In accordance with this finding many studies have demonstrated that, in the absence of renal failure, HDL-C and apoA-I are often higher in subjects with type 1 diabetes than in the control population [27,28]. It has been indicated that patients with type 1 diabetes have greatly increased phospholipid transfer protein (PLTP) activity [29], and higher PLTP activity was associated with more circulating apoA-I levels [28]. Thus, it seems that higher PLTP activity makes an important contribution to the higher apoA-I levels and altered HDL subclass distribution in type 1 diabetes [28].

Few studies have investigated the effects of exercise training on circulating apoA-I levels in subjects with type 1 diabetes mellitus. Lehmann et al. [30] reported that 3 month increased of physical activity from 195 ± 176 to 356 ± 164 min associated with enhanced plasma apoA-I levels in subjects with type 1 diabetes mellitus [30]. Also, increased of circulating apoA-I and HDL levels was found in young men with type 1 diabetes mellitus after 12–16 week endurance exercise program [31]. To our knowledge, in the present study, for the first time we shown that 4 weeks of resistance training was associated with higher plasma apoA-I and HDL levels in type 1 diabetic rats compared with control group. The underlying mechanisms responsible for alterations in circulating apoA-I levels due to exercise training is poorly understood. It is indicated that adiponectin enhanced apoA-I synthesis in the liver [32]. Although in this study we did not assessed plasma adiponectin concentrations, increased of circulating adiponectin levels reported in several studies due to exercise training [33,34]. Also, increase of ABCA1 expression, and ApoA-I and some other factors involved in the process of cholesterol reverse transport such as lecithin cholesteryl acyl transferase (LCAT), cholesteryl ester transfer protein (CETP), PLTP and Scavenger receptor BI (SR-BI) have been suggested the main mechanisms of increasing HDL-C [35].

While several studies have examined the effects of FABP4 expression on adiposity, insulin resistance, metabolic syndrome, and type 2 diabetes, there are only limited data available regard in type 1 diabetes mellitus. Previous studies indicated that circulating FABP4 levels are considered to be a link between obesity, insulin resistance, diabetes, and cardiovascular diseases [10]. Moreover, some of studies shown that circulating FABP4 levels were increased in overweight and obese subjects compared with lean controls, and was associated with markers of insulin resistance and obesity [10]. While weight reduction associated with decline of circulating FABP4 levels [36]. In accordance with these studies, we found that lower plasma FABP4 levels in STZ-induced diabetic rats accompanied with significant weight reduction.

Although high levels of circulating FABP4 has been suggested as a biomarker of metabolic syndrome [9], controversial results found in chronic pancreatitis [17], and lean type 1 diabetic patients [18]. Also, Hsu et al. [37] did not find any significant differences in serum FABP4 levels between type 1 and type 2 diabetic patients [37]. In the present study, decreased of plasma FABP4 level was associated with hyperglycemia induced by STZ injection. It seems that alterations in body weights to be an effective factor in contradictory observations in previous studies. Significant differences in circulating FABP4 concentrations were found in studies that significant differences in body weights between groups has been found. On the other hand, high levels of blood glucose and inflammatory markers were not always associated with increased circulating FABP4 levels [37,38]. Also, plasma FABP4 in obese type 1 diabetic patients were significantly higher than in non-obese type 1 diabetic patients [18]. Thus, it seems that adipocyte hypertrophy induced by weight gain and adiposity result in elevation of FABP4 expressions and subsequent release into the bloodstream. In this regard, Skurk et al. suggested that adipocyte size is an important determinant of adipokine secretion [39]. However, further studies are needed in order to determine the main factors affecting circulating FABP4 levels.

Few studies have investigated the effects of exercise training on circulating FABP4 levels. After 3 months of the exercise training in obese women, serum FABP4 levels decreased significantly along with a reduction in body weight, BMI, waist circumference, fasting glucose and total cholesterol levels [40]. Also, Lázaro et al. [41] indicated that increasing aerobic physical activity can decrease plasma FABP4 levels, independently of weight reduction in patients with cardiovascular risk [41]. While, FABP4 gene expression was significantly elevated after 10 weeks of aerobic training in the epididymal and retroperitoneal adipose tissues of Obese Zucker rats. Moreover, Fischer et al. [42] found that FABP4 mRNA and protein expressions were much higher in endurance trained compared to control individuals [42]. In the present study trained diabetic rats have a higher plasma FABP4 levels than diabetic control group. Moreover, this exercise training program leads to preventing of weight reduction induced by diabetic induction. Thus, it seems this training program with inhibiting weight reduction have a regulatory function for circulating FABP4 levels. Interestingly, we found positive correlation between plasma FABP4 levels and muscle and body weight in diabetic rats. On the other hand, previous studies indicated that exercise training result in significant increase in muscle FABP4 levels [38]. Thus, in the present study higher plasma FABP4 levels in trained rats could be partly due to the influence of resistance training on muscle FABP4 contents and releasing in bloodstream.

We did not find any significant changes in plasma lipid profile and liver cholesterol levels after 4 week resistance training despite enhanced plasma FABP4 levels. However, liver triglyceride levels in trained groups were significantly lower than control groups. In accordance with this finding, previous studies indicated that resistance training could reduce liver fat content [43]. For example, significant reduction of liver fat was observed in patients with non-alcoholic fatty liver after 8 week resistance training a long with improve in glucose metabolism [44]. Moreover similar results found in studies with animal models. In ovariectomized or normal rats 12 weeks resistance training decreased muscle and liver lipid contents [22,45]. Also, improved serum lipid profile was observed in rats fed with normal or high-fat diets after 8 weeks of resistance training [24]. Thus, it seems that relatively short duration of resistance training program used in this study compared with previous studies, and low energy consumption induced by this type of training compared with aerobic training could be reasons for no significant changes in circulating lipid profile.

## Conclusions

These results indicate that resistance training increases serum apoA-I and FABP4 concentrations in streptozotocin-induced diabetic rats. Inhibiting role of resistance training in weight loss induced by diabetic induction may be one of effective mechanisms for this observation. However, further research is needed to elucidate physiological significance of circulating FABP4 levels.

## Competing interests

The authors declare that they have no competing interests.

## Authors’ contributions

AS conception and design of study, collection and possession of data, statistical analysis and drafted the initial manuscript, ETG participated in the study design, data analysis and revision of the manuscript. Both authors read and approved the final manuscript.
